# Bronchoscopy and the Pulmonologist in Contemporary Lung Cancer Care

**DOI:** 10.3390/cancers18152459

**Published:** 2026-07-31

**Authors:** Alireza Nathani, Jack Inglis, H. Erhan Dincer

**Affiliations:** 1 University of Minnesota Medical School, University of Minnesota, Minneapolis, MN 55455, USA; 2 Hennepin County Medical Center, Minneapolis, MN 55455, USA; jack.inglis@hcmed.org

**Keywords:** pulmonologist, lung cancer, bronchoscopy, diagnosis

## Abstract

This review examines the expanding role of the pulmonologist in the care of patients with lung cancer, including diagnosis, mediastinal staging, tissue stewardship, therapeutic bronchoscopy, and involvement in longitudinal pulmonary care. A multidisciplinary approach is essential in providing high-quality patient care. Emerging strategies include structured nodule programs, mobile health screening, and artificial intelligence to improve the timely diagnosis of lung cancer.

## Simple Summary

Bronchoscopy plays an important role in diagnosing and staging lung cancer and obtaining sufficient tissue to guide treatment. This review discusses advances in bronchoscopic diagnosis and staging, factors that influence the success of tissue acquisition, and the pulmonologist’s role in selecting the diagnostic approach best suited for each patient. It also highlights the importance of multidisciplinary physician coordination, therapeutic bronchoscopy for central airway obstruction, and ongoing pulmonary care throughout the patient’s lung cancer journey. Additionally, we discuss emerging strategies to improve early detection, including lung nodule programs, mobile low-dose imaging screening platforms, and the introduction of artificial intelligence and radiomics.

## 1. Introduction

Modern lung cancer evaluation is no longer limited to establishing whether malignancy is present. The diagnostic pathway must define the anatomic extent of disease, establish histology, preserve sufficient tissue for biomarker testing, and minimize procedural risk. Inadequate tissue sampling for staging or molecular profiling may lead to treatment delays, additional diagnostic procedures, and suboptimal therapeutic decision-making.

As technological advances have expanded the capabilities of bronchoscopy, the pulmonologist has become increasingly pivotal to thoracic oncology care. Endobronchial and esophageal endosonography now anchor minimally invasive mediastinal and hilar staging, while radial ultrasound, navigational platforms, robotic bronchoscopy, and intraprocedural imaging have broadened access to peripheral pulmonary lesions that historically often required transthoracic approaches. Procedural success now depends on more than lesion access alone; it requires individualized procedural planning, real-time adequacy assessment, judicious specimen handling and processing, and early coordination with pathology and oncology. The pulmonologist’s contribution also extends beyond diagnosis: therapeutic bronchoscopy, longitudinal pulmonary care, and management of treatment-related complications position the pulmonologist as an integral member of the thoracic oncology team throughout the course of disease.

This review examines the evolving landscape of lung cancer evaluation across five domains: mediastinal and hilar staging, bronchoscopic evaluation of peripheral pulmonary lesions, procedural determinants of safety and tissue adequacy, multidisciplinary thoracic oncology care, and future directions aimed at improving early detection, procedural accuracy, and equitable access to timely diagnosis.

## 2. Mediastinal and Hilar Staging

Accurate mediastinal and hilar staging is central to the management of suspected or confirmed non-small cell lung cancer. Nodal status informs prognosis and often determines the therapeutic pathway, including upfront surgical resection, neoadjuvant or perioperative systemic therapy as part of a surgery-based approach, definitive chemoradiation, or primarily systemic therapy. Contemporary TNM9/IASLC (Tumor, Node, Metastasis/International Association for the Study of Lung Cancer) staging now distinguishes single-station N2 disease (N2a) from multistation N2 disease (N2b), reflecting the prognostic and therapeutic importance of nodal extent [1,2]. Although computed tomography (CT) and positron emission tomography/computed tomography (PET/CT) are essential components of initial clinical staging, imaging alone is insufficiently accurate to reliably confirm or exclude mediastinal nodal metastases. The American College of Chest Physicians (ACCP) guidelines report approximate sensitivities and specificities of 55% and 81% for CT and 77% and 86% for PET in identifying mediastinal nodal disease, emphasizing that tissue confirmation is often required when nodal status would alter management [3]. Contemporary European Society for Medical Oncology (ESMO) guidance aligns with this principle, recommending CT and PET or PET/CT as the initial imaging framework and invasive endosonographic staging when mediastinal or hilar nodes are abnormal on imaging [1].

Multiple guidelines therefore support a “needle-first” endosonographic approach when invasive mediastinal staging is indicated. The ACCP recommends endobronchial ultrasound-guided transbronchial needle aspiration (EBUS-TBNA), endoscopic ultrasound-guided needle aspiration (EUS-NA), or combined EBUS/EUS as the preferred method in patients with high suspicion for N2/N3 disease [3]. The revised European Society of Thoracic Surgeons (ESTS) guidelines similarly identify EBUS/EUS-NA as the first choice for tissue confirmation in patients with CT-enlarged (generally defined as >1 cm) or PET-positive mediastinal nodes when expertise is available [4]. The European Society of Gastrointestinal Endoscopy/European Respiratory Society/European Society of Thoracic Surgeons (ESGE/ERS/ESTS) guideline recommends endosonography over surgical staging as the initial procedure in patients with suspected or proven non-small cell lung cancer and abnormal mediastinal or hilar nodes on CT and/or PET, and ESMO likewise recommends needle aspiration under EBUS and/or EUS guidance for pathological assessment of suspicious nodes [1,5].

**Figure 1:**
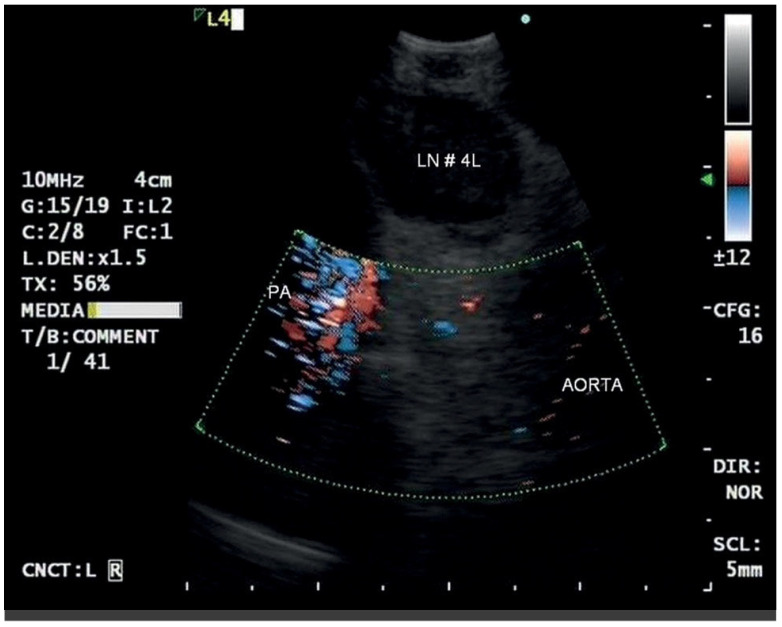
The left lower paratracheal node (Station 4L), pulmonary artery (PA), and aorta (AO) are indicated in the figure.

Within an endosonography-first approach, EBUS-TBNA serves as the primary bronchoscopic modality for mediastinal and hilar staging. It allows real-time ultrasound-guided sampling of nodal stations adjacent to the tracheobronchial tree, including paratracheal stations 2R, 2L, 4R, and 4L; subcarinal station 7; hilar station 10; and interlobar/lobar stations 11 and 12 [4,5,6], as shown in Figure 1. EBUS can visualize and sample nodes with a short-axis diameter as small as 5 mm via ultrasound. During systematic staging, respective nodes meeting the sampling threshold should be assessed in each relevant station, with particular attention to the largest or most suspicious node [4]. This reach is particularly valuable for pulmonologist-led staging, since diagnosis and nodal staging can often be accomplished during the same minimally invasive procedure, and appropriately processed specimens may also support immunohistochemistry and molecular testing [1,6].

Evidence consistently supports EBUS-TBNA as an accurate and safe modality for mediastinal nodal staging, although performance varies with patient selection and the quality of the procedure. In a systematic review and meta-analysis of 11 studies including 1299 patients, Gu et al. reported a pooled sensitivity of 0.93 and specificity of 1.00 for EBUS-TBNA in mediastinal nodal staging, with only two major complications reported across the cohort [7]. The diagnostic performance of EBUS-TBNA also depends on pretest selection: Sensitivity was higher when staging was directed by suspicious CT or PET findings than when it was performed in patients without such findings (0.94 versus 0.76, respectively) [7]. Additional procedural factors, including operator experience, systematic station assessment, number of passes, and cytopathology support, may also affect performance. Rapid on-site evaluation (ROSE), in which cytology staff assesses specimen adequacy during the procedure, has not consistently improved overall diagnostic yield in meta-analysis, but it may reduce the number of needle passes and need for subsequent bronchoscopy [8].

Proper EBUS staging requires more than sampling the largest or most PET-avid node. To reduce the possibility of needle carryover causing false upstaging, nodal sampling should proceed from the most prognostically advanced stations toward those closer to the primary tumor. If a single needle is used, this generally means sampling N3 stations before N2 and N1 stations [4,6]. Thus, for a left-sided lung cancer, suspicious or accessible contralateral right-sided mediastinal or hilar nodes should be evaluated before ipsilateral left-sided N2 or N1 stations; for a right-sided primary tumor, suspicious contralateral left-sided stations are sampled first [4,6]. Systematic staging also requires deliberate station assessment rather than purely targeted biopsy. When CT or PET suggests mediastinal involvement in non-small cell lung cancer, the ESGE/ERS/ESTS guideline favors a systematic nodal evaluation rather than selective sampling alone, recommending samples from at least 4R, 4L, and 7 [5]. Accurate station labeling remains essential because the distinction between N1, N2, and N3 disease—and now between N2a and N2b disease—directly affects treatment recommendations and multidisciplinary assessment of resectability [1,2,9].

Because EBUS does not access all relevant nodal stations, esophageal endosonography (EUS) extends the reach of minimally invasive staging. EBUS is best suited for nodes adjacent to the central airways, whereas esophageal ultrasound-guided sampling can access nodal stations and anatomic regions that are technically challenging to reach bronchoscopically. This can be performed either with a conventional gastrointestinal echoendoscope, referred to as EUS-FNA, or by passing the EBUS bronchoscope into the esophagus, referred to as EUS-B-FNA. These approaches provide reliable access to lower mediastinal stations 8 and 9 and station 7; they can also reach left-sided paratracheal station 4L, where EUS may offer a technical advantage over EBUS for small or difficult-to-sample nodes [5]. EUS can also evaluate selected subdiaphragmatic sites, including the left adrenal gland and left liver lobe, although transgastric left adrenal biopsy using the EBUS scope is considered experimental by the ESGE/ERS/ESTS guideline [5]. For pulmonologists, EUS-B is particularly relevant because it allows the same operator to extend staging beyond the airway-accessible stations and perform a more comprehensive endosonographic examination in one session.

Combined endosonography is supported by both pooled diagnostic-performance studies and randomized trial data. The combined use of EBUS and EUS/EUS-B expands the reach of minimally invasive staging and increases the sensitivity and thoroughness of the evaluation compared with either modality alone [5,10]. In a meta-analysis of combined EBUS-TBNA and EUS-FNA, Zhang et al. reported a pooled sensitivity of 0.86, specificity of 1.00, and an area under the curve of 0.99 [10]. In the ASTER trial, participants with operable non-small cell lung cancer in whom mediastinal sampling was indicated were allocated to either upfront surgical staging or an endosonography-first pathway, with surgical confirmation reserved for cases where endosonography was negative. Sensitivity for mediastinal nodal metastases was 79% with surgical staging alone, 85% with endosonography alone, and 94% with endosonography followed by surgical staging. The endosonography-first strategy also reduced unnecessary thoracotomies from 18% to 7% [11]. These findings helped establish an endosonography-first approach, often combining EBUS and EUS, as the preferred initial invasive staging strategy while preserving a role for surgical confirmation in selected high-risk patients.

Cervical mediastinoscopy was previously considered the gold standard for invasive mediastinal staging, but its role has evolved in the era of high-quality endosonography. Mediastinoscopy remains highly specific and provides surgical tissue from superior mediastinal stations, particularly 2R, 2L, 4R, 4L, and 7 [3,4]. However, it requires general anesthesia, is more invasive than endosonography, and does not access hilar stations or inferior mediastinal stations 8 and 9. Current guidelines generally position mediastinoscopy as a confirmatory or complementary procedure rather than the default first invasive test. ACCP, ESTS, and ESGE/ERS/ESTS guidelines recommend needle-based endosonographic techniques first when expertise is available, while advising surgical staging after a negative needle result if clinical suspicion remains high [3,4,5].

The need for routine confirmatory mediastinoscopy after negative systematic endosonography is increasingly debated. In the MEDIASTrial, non-small cell lung cancer patients who underwent systematic endosonography without evidence of nodal metastases were assigned to either proceed directly to lung resection or first undergo confirmatory mediastinoscopy. Despite detecting nodal metastases in 8.0% of cases, mediastinoscopy-first staging did not significantly reduce unforeseen N2 disease compared with immediate resection, with rates of 7.7% and 8.8%, respectively [12]. The benefit of mediastinoscopy-first staging was most apparent in patients with cN1 disease, in whom unexpected N2 involvement was found less often than after immediate resection (7.0% versus 13.6%) [12]. While guidelines still generally recommend surgical confirmation of negative endosonography in high-risk cases, these data suggest confirmatory mediastinoscopy may be omitted in selected patients following high-quality systematic endosonographic evaluation [3,4,5,12]. Mediastinoscopy remains important when endosonography is inadequate, technically limited, discordant with imaging, or when residual suspicion for mediastinal disease remains high [3,4,5,12].

Video-assisted thoracoscopic surgery (VATS) also has a limited but important complementary role in lung cancer staging. VATS is not a routine first-line mediastinal staging modality in modern endosonography-centered pathways, but it can clarify T, N, or M stage in selected cases. It may identify occult pleural disease, evaluate suspicious pleural effusions or pleural implants, assess chest wall or mediastinal invasion, and obtain tissue when less invasive approaches are insufficient [13]. VATS is also relevant for aortopulmonary window nodes. Stations 5 and 6 may be visualized by EUS but are rarely sampled safely because of proximity to major vascular structures; surgical staging, including VATS, is often used for these stations when pathologic assessment would alter management [3,5]. Because VATS requires general anesthesia and single-lung ventilation, it cannot provide access to contralateral N3 nodes, and it may have reduced utility in patients with pleural adhesions; it is best framed as a selective surgical adjunct rather than a first-line alternative to EBUS/EUS-based staging [4,13].

Modern mediastinal and hilar staging highlights the expanding role of the pulmonologist in lung cancer diagnosis. EBUS-TBNA, complemented by EUS and/or EUS-B when appropriate, allows the pulmonologist to integrate imaging interpretation, systematic nodal staging, and tissue acquisition within a single diagnostic strategy. In contemporary practice, acquired tissue must often support not only histologic diagnosis but also biomarker testing when clinically indicated [1]. This reflects a broader shift in lung cancer care, in which treatment selection increasingly depends on both nodal burden and molecular status. As neoadjuvant, perioperative, adjuvant, and consolidation strategies continue to evolve, the quality of the initial invasive staging procedure has become increasingly consequential.

## 3. Bronchoscopic Evaluation of Peripheral Pulmonary Lesions

The widespread adoption of chest CT and the implementation of lung cancer screening programs have substantially increased the detection of small peripheral pulmonary nodules. Data from a large integrated health system illustrate the scale of this shift: By 2012, newly identified pulmonary nodules occurred at a rate of 6.6 per 1000 person-years, up from 3.9 per 1000 person-years in 2006, corresponding to an estimated 1.57 million affected adults in the United States each year [14]. Most ultimately prove benign, but a subset requires tissue diagnosis because of clinical risk, radiographic growth, PET avidity, or planned definitive therapy. These lesions pose a distinct challenge: they lie beyond direct endobronchial visualization, so successful biopsy depends on accurate lesion localization, confirmation that the sampling tool has reached the target, and tissue sufficient for histologic and molecular characterization.

Tissue diagnoses of peripheral pulmonary lesions can be pursued through three broad routes: bronchoscopic biopsy, CT-guided transthoracic needle biopsy, and surgical biopsy or resection. Historically, flexible bronchoscopy demonstrated limited sensitivity, especially for smaller lesions. Earlier guideline data reported a sensitivity of 34% for lesions under 2 cm [15]. Ultrathin bronchoscopy was developed to extend reach into smaller distal airways and may improve access for selected difficult-to-access lesions, though it has remained largely adjunctive [16]. CT-guided transthoracic needle biopsy offered a higher-yield alternative, particularly for pleural-based or anatomically favorable lesions, but at the cost of greater morbidity; in a systematic review and network meta-analysis of 363 studies and 79,519 nodules, it achieved the highest pooled diagnostic yield among nonsurgical modalities while also carrying the highest rates of pneumothorax and clinically significant bleeding [17]. Surgical biopsy or resection via VATS can serve a dual diagnostic and therapeutic role and is appropriate in surgical candidates with a nondiagnostic nonsurgical biopsy or a probability of malignancy sufficient to warrant definitive management [3]. More recent bronchoscopic advances have since narrowed the yield and safety gap with transthoracic biopsy, making bronchoscopy an increasingly viable, reliable, and first-line diagnostic option for selected peripheral lesions.

**Figure 2:**
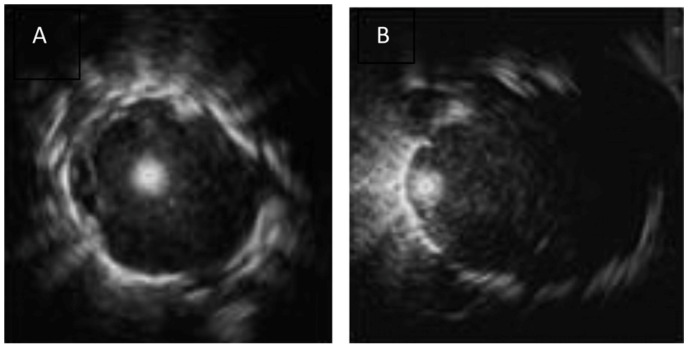
Concentric radial endobronchial ultrasound (EBUS) image of a lesion with the probe centered and surrounded by the lesion (**A**). Eccentric radial EBUS image of a lesion with the probe positioned at the edge of the lesion (**B**).

Radial probe endobronchial ultrasound (rEBUS) represented an early and important step in this evolution, allowing bronchoscopists to localize peripheral lesions from within the airway. Rather than relying on two-dimensional fluoroscopic projection, rEBUS provided real-time ultrasound confirmation that the probe had reached the lesion or its immediate vicinity. The ultrasound image produced is either concentric, which means the probe is directly inside the lesion, or eccentric, indicating the lesion is adjacent to the lesion [18], as shown in Figure 2. In a large retrospective study, rEBUS localized nearly all peripheral lesions (96%), but diagnostic yields depended strongly on probe position: The overall yield was 69%, reaching 84% when the radial probe was visualized within the lesion compared with 48% when it was adjacent [18]. A meta-analysis supports rEBUS as a safe localization tool, with a pooled malignancy sensitivity of approximately 72% and a pneumothorax rate of less than 1%, although heterogeneity and possible publication bias limit confidence in these estimates [19]. While rEBUS remains a useful adjunct, it is limited by the fact that the radial probe cannot independently steer the bronchoscope and must be removed before biopsy instruments can be advanced, leaving the challenges of peripheral navigation and real-time confirmation of the tool’s position during sampling unresolved.

Virtual bronchoscopic navigation and electromagnetic navigation bronchoscopy (ENB) were developed to address this navigational constraint. Virtual bronchoscopic navigation uses CT-derived airway reconstructions to plan the bronchial route to a peripheral target; in a randomized trial, pairing it with rEBUS improved diagnostic yields over rEBUS alone and shortened procedure times [20]. ENB built on this by incorporating real-time electromagnetic tracking of instrument position relative to a preprocedural CT map, allowing the operator to guide an extended working channel or locatable instrument toward the lesion. In the prospective multicenter NAVIGATE study, ENB achieved a 12-month diagnostic yield of 73% and malignancy sensitivity of 69%, while clinically significant pneumothorax occurred in less than 3% of cases. The frequent discretionary use of rEBUS, reported in 57% of cases, underscores its role as a complementary localization tool [21]. Pooled estimates across successive generations of guided bronchoscopy show diagnostic yields holding near 70%, with lesion size greater than 2 cm and a visible bronchus sign being the most consistent predictors of success [22]. While these platforms improved route planning, they remained limited by the reach and stability of conventional flexible bronchoscopes in the distal airways.

Robotic-assisted bronchoscopy represents the most recent advancement in navigational bronchoscopy, extending distal reach and mechanical stability into airways that earlier platforms could not reliably access. These systems use a steerable robotic catheter, controlled externally by the operator and guided by a preprocedural CT-based airway map to advance biopsy instruments into distal bronchial segments. Reported outcomes vary substantially by study design and diagnostic-yield definition. In a 2025 meta-analysis of 27 cohort studies, pooled yields ranged from approximately 70% by strict criteria to 87% by intermediate criteria, with a complication rate of 3.0% [23]. This definition-dependent variability was also illustrated by the prospective multicenter TARGET trial: Among 679 patients evaluated with the Monarch platform, the diagnostic yield was 61.6% using strict ATS/CHEST criteria and 83.2% using investigator-reported criteria [24]. In the RELIANT randomized trial, robotic bronchoscopy was noninferior to ENB, with target tissue obtained at similar rates (78% versus 76%, respectively) and no significant difference in complications [25], supporting robotic bronchoscopy as a capable alternative to ENB with potential advantages in distal reach. While robotic bronchoscopy has resulted in navigational improvements, diagnostic yields remain limited by the separate challenge of confirming the tool-in-lesion position at the time of sampling.

CT-to-body divergence, the mismatch between a lesion’s position on the preprocedural CT and its actual intraprocedural location, can result from changes in lung volume, respiratory motion, patient positioning, or atelectasis [26]. For small nodules, even minor displacement may place the biopsy tool outside the target. Several intraprocedural imaging methods have been developed to counter this. Digital tomosynthesis reconstructs depth-resolved images from multiple low-dose X-ray projections acquired during the procedure; cone-beam CT (CBCT) uses a rotating flat-panel detector to generate intraprocedural volumetric images comparable to conventional CT within the procedure suite; augmented fluoroscopy overlays CT-derived lesion maps onto live fluoroscopic imaging to guide tool positioning. Together, these tools update target localization and allow confirmation of tool-in-lesion position across a range of navigational platforms. In an early retrospective study, digital tomosynthesis-corrected ENB improved yield over standard ENB (79% vs. 54%), with both tomosynthesis correction and a positive rEBUS view independently associated with diagnostic success [26]. A systematic review of CBCT combined with rEBUS reported a pooled yield of 80% with low complication rates, although certainty was limited by heterogeneity and predominantly observational data [27]. In a single-center retrospective study, adding CBCT integration to shape-sensing robotic bronchoscopy was associated with higher diagnostic yield and accuracy, despite smaller and more peripheral lesions in the CBCT cohort [28]. These findings suggest that further gains in peripheral bronchoscopy may depend less on navigation alone than on reliable intraprocedural confirmation and re-targeting.

The evidence supports a shift from viewing peripheral bronchoscopy as a single diagnostic procedure to understanding it as an integrated strategy that combines navigation, lesion localization, intraprocedural confirmation, and optimized tissue acquisition. The VERITAS trial illustrates how this approach has narrowed the historical gap between bronchoscopic and transthoracic biopsy, with navigational bronchoscopy using digital tomosynthesis demonstrating noninferior diagnostic accuracy to CT-guided biopsy for 10–30 mm peripheral nodules, similar diagnostic yield, and a significantly lower pneumothorax rate in the primary safety analysis [29]. Contemporary American Association of Bronchology and Interventional Pulmonology (AABIP) guidance reflects this evolving evidence base, suggesting consideration of peripheral navigational bronchoscopy over CT-guided transthoracic biopsy for indeterminate pulmonary nodules while accounting for patient factors, nodule characteristics, local expertise, and cost [30]. CT-guided biopsy and surgical resection remain important alternatives when they offer greater diagnostic efficiency, staging value, or feasibility. Technology has expanded the options available for peripheral sampling; selecting among them requires matching the approach to the patient’s clinical context, with the goal of obtaining tissue that is safe to acquire, sufficient for diagnosis, and adequate for molecular characterization.

## 4. Procedural Determinants of Safety, Yield, and Tissue Adequacy

In modern lung cancer evaluation, the availability of bronchoscopic, transthoracic, and surgical sampling approaches has shifted the central procedural question from whether tissue can be acquired to whether the selected strategy can provide the diseased tissue, from the right target, with an acceptable risk profile. A successful biopsy must align the sampling route with lesion anatomy and patient-specific risk, obtain tissue sufficient for histologic classification, and preserve enough appropriately processed material for clinically indicated biomarker testing. These goals do not always align: The highest-yield approach may not be the safest for a given patient, and a specimen sufficient to diagnose malignancy may not contain enough material for molecular profiling. Balancing these goals requires integrating several determinants of procedural success on an individualized basis: lesion characteristics, patient factors that dictate procedural safety, sampling strategy, real-time adequacy assessment, and tissue stewardship [30,31].

Lesion characteristics determine both diagnostic yield and the need for invasive mediastinal staging. Across guided-bronchoscopy series, larger lesion size and a visible bronchus sign are consistently associated with higher diagnostic yield, whereas smaller lesions and those lacking a clear airway path remain more challenging to sample successfully [22]. Lesion location may impact procedural difficulty, particularly when lower-lobe or more mobile targets are affected by respiratory motion, atelectasis, or CT-to-body divergence; however, location has not been consistently predictive of yield [26]. Radiographic phenotype informs both expected yield and staging strategy. In network meta-analysis, solid lesions achieved higher diagnostic yield than subsolid lesions when sampled via rEBUS and ENB [17]. Pure ground-glass nodules and part-solid lesions with a consolidation-to-tumor ratio of 0.5 or less carry very low nodal metastatic risk and generally do not warrant invasive mediastinal staging, whereas tumors larger than 3 cm and lesions with a greater solid component raise concern for occult mediastinal metastasis and may favor invasive staging even when the hilum and mediastinum appear radiographically normal [30]. While lesion-level features help define the optimal diagnostic target and anticipated yield, the final sampling route must also account for patient-specific anatomy and physiologic risk.

Patient anatomy, physiology, and comorbidities can make a technically feasible biopsy route clinically unfavorable [30,31]. For transthoracic biopsy, risk is shaped not only by lesion accessibility but also by the needle path: emphysematous lung, crossing bullae or fissures, multiple pleural punctures, greater lesion depth, and small or non-pleural-contacting lesions all increase pneumothorax risk [32]. These factors are especially consequential in patients with limited pulmonary reserve, severe emphysema, frailty, or poor performance status, in whom even a usually manageable pneumothorax may cause clinically meaningful decompensation. Bronchoscopic biopsy avoids intentional pleural traversal and can combine parenchymal sampling with EBUS staging, but it introduces different constraints, including tolerance of airway manipulation, procedure duration, and sedation or anesthesia requirements [30,31]. Bleeding risk is another important consideration: Clinically significant bleeding is reported more often with CT-guided transthoracic biopsy than with guided bronchoscopic techniques, and bronchoscopic bleeding, when encountered, can often be managed endobronchially [17]. The inability to tolerate prone or lateral positioning, breath-holding, or post-biopsy positioning may make CT-guided biopsy less suitable, whereas severe hypoxemia, airway instability, or inability to tolerate prolonged sedation may favor a shorter percutaneous procedure or biopsy of an alternative site [31,32]. Thus, patient-level factors can alter both the likelihood and clinical consequences of route-specific complications, requiring procedural selection to balance expected tissue yield against the patient’s physiologic reserve and ability to tolerate adverse events.

Navigation and localization are necessary but not sufficient; once the target is reached, diagnostic success depends on keeping the sampling catheter or guide sheath aligned with the lesion and obtaining representative tissue suitable for histologic and molecular evaluation. The 2025 AABIP guideline supports considering a multi-tool sampling strategy rather than reliance on a single instrument (e.g., needle) when bronchoscopic sampling of peripheral pulmonary lesions is pursued. In its meta-analysis of ten studies including 1572 patients, multi-tool sampling improved diagnostic yield compared with single-tool sampling (odds ratio 1.78; 95% CI, 1.49–2.14) without a corresponding rise in complications [30]. In practice, tool selection should be individualized rather than simply additive: Transbronchial needle aspiration, forceps biopsy, brushings, and lavage can provide complementary tissue or cytologic material, and the optimal combination depends on lesion size, airway relationship, access trajectory, bleeding risk, molecular testing needs, and operator expertise. For selected peripheral lesions in which conventional forceps sampling may provide limited or artifact-prone tissue, transbronchial cryobiopsy can increase specimen size and quality, although its use depends on airway access, bleeding risk, and local expertise. In a randomized comparison, cryobiopsy improved diagnostic yield over forceps biopsy (75.9% vs. 48.1%) and provided larger, higher-quality samples for subsequent molecular profiling [33]. Even after the initial sampling, procedural success still depends on real-time assessment of whether the material being obtained is sufficient for the intended diagnostic purpose.

Rapid on-site evaluation (ROSE) refers to immediate cytologic assessment of sampled material during the procedure, typically by preparing a direct smear from aspirated material or touch preparations for small core tissue biopsies, rapidly staining it, and having cytopathology personnel review it on-site or by telepathology while sampling is still underway. This real-time feedback can confirm whether representative material has been obtained; indicate whether additional passes or alternative tools are needed; and guide allocation of limited tissue across cytology, cell block, histology, and molecular testing [30]. In the AABIP meta-analysis of six studies including 2710 patients, use of ROSE during bronchoscopic sampling of peripheral pulmonary lesions was associated with higher diagnostic yield (odds ratio 2.71; 95% CI, 2.06–3.58) without significantly prolonging the procedure, though the guideline appropriately conditions its use on local cytopathology resources rather than recommending it universally [30]. In mediastinal nodal staging, ROSE may reduce needle passes or confirm adequacy, but it has not consistently improved overall yield and should not displace systematic station sampling. The College of American Pathologists (CAP) guideline frames ROSE as a tool for both adequacy assessment and deliberate specimen allocation for ancillary studies [34]. Of note, a preliminary diagnostic result should not automatically end sampling if additional material is needed for cell block preparation, immunohistochemistry, or molecular analysis.

Molecular adequacy has become a defining standard for lung cancer tissue acquisition. A biopsy that establishes the diagnosis of non-small cell lung cancer may still fail to support additional clinically indicated biomarker testing. This shortfall should not be attributed reflexively to route choice: Available comparative data have not shown a clear molecular-adequacy advantage for either guided bronchoscopy or CT-guided transthoracic biopsy [30,31]. Adequacy is instead shaped by lesion-dependent factors, such as tumor cellularity and necrosis, as well as modifiable factors, including tool choice, pass quality, specimen preparation, and tissue conservation. The CAP guideline recommends obtaining at least three cores when transthoracic core biopsy is used, fixing core specimens in 10% neutral-buffered formalin, and preparing a cell block when only fine-needle aspiration is performed [34]. ESMO guidance emphasizes tissue conservation after sampling, which includes minimizing unnecessary sectioning of tissue blocks and a morphology-first approach to non-small cell lung cancer subtyping, with diagnostic immunohistochemistry often limited to p40 and thyroid transcription factor-1 (TTF-1) testing when morphology is insufficient [1]. These steps help preserve residual tumor for programmed death-ligand 1 (PD-L1) testing and DNA/RNA-based molecular profiling [1,34]. Tissue adequacy is therefore a product of the entire acquisition pathway, including target selection, tool choice, pass strategy, fixation, specimen triage, and early communication of testing needs, rather than the biopsy route alone.

Liquid biopsy is a complementary tool to bronchoscopic biopsy and should not replace tissue acquisition. Plasma-based circulating tumor DNA analysis can identify genomic alterations. Tissue remains necessary for histologic diagnosis; assessment of PD-L1 expression, excluding alternative diagnoses; and staging. Most importantly, because the sensitivity of liquid biopsy is dependent on the amount of circulating DNA, a negative liquid biopsy cannot exclude a genetic alteration and should be followed by tissue-based gene sequencing [35].

Collectively, the determinants discussed above converge on a single procedural goal: maximizing the diagnostic value of the first biopsy. Achieving this requires matching modality to lesion and patient, anticipating route-specific complications, combining sampling tools when appropriate, using ROSE where it adds value, and preserving material for downstream testing. The intended result is a biopsy strategy that is safe, stage-informative, and molecularly adequate. A nondiagnostic or molecularly insufficient first biopsy is not a neutral outcome; it may delay staging and treatment planning, consume resources, and expose the patient to another procedure. These procedural considerations therefore provide the foundation for multidisciplinary planning, in which biopsy target, procedural sequence, tissue handling, and treatment readiness are coordinated across the thoracic oncology team.

## 5. Bronchoscopy in Multidisciplinary Cancer Care

Lung cancer care has become more complex because treatment depends on an integrated assessment of anatomy, lymph node staging, histology, molecular markers, and the patient’s ability to undergo therapy. In a systematic review and meta-analysis, Stirling et al. found that multidisciplinary review was associated with improvements in diagnostic and staging processes and, in some studies, overall survival [36].

Pulmonologists are often the first point of contact after an incidental nodule or lung cancer screening CT is performed [37]. Additionally, pulmonologists will see referrals for lung masses, mediastinal abnormalities, or pleural diseases. This initial contact is critical as the initial diagnostic strategy can influence procedural morbidity, effectiveness of tissue sampling, complications, and subsequent cancer course. The goal is not to simply select a procedure but rather to select the correct one that will lead to the fewest complications and adequate tissue acquisition for molecular testing, all while minimizing delays in care.

The optimal biopsy strategy is clinically determined and varies depending on the level of experience and training the pulmonologist has. There are fellowship-trained interventional pulmonologists, advanced bronchoscopists, and general pulmonologists who can perform bronchoscopy. Referral should prioritize physicians who regularly perform bronchoscopic lung biopsies, participate in ongoing quality review, and have access to the technology needed to address the clinical question. Ali MS et al. discovered, through a systematic review of 1779 lung lesions, a pooled diagnostic yield of 84.3% for robotic-assisted bronchoscopy for lesions less than 2 cm [38]. Advances in technology have increased the specificity and sensitivity of diagnosing peripheral pulmonary lesions; however, not all pulmonologists have access to the most up-to-date tools. Selection should account for both operator experience and the technologies needed for the case, which include a robotic platform and cone-beam CT. Although these are not the only important factors in obtaining a biopsy, research by Bashour SI et al. has shown that integration of a robotic platform and cone-beam CT helps compensate for CT-to-body divergence, allowing intraprocedural conformation of the tool in the lesion [39].

The pulmonologist’s contribution extends beyond making the diagnosis. Accurate and thorough mediastinal staging is one of the most important steps in thoracic oncology as it determines future treatments. Historically, this has been performed through surgical techniques that require a skin incision, such as mediastinoscopy. Recent advancements in ultrasound technology have made the standard of care linear endobronchial ultrasound (EBUS) [40]. The difference between a thoroughly performed EBUS and one that targets the largest or PET-avid node can result in significant treatment change [41].

The advancement of thoracic oncology has resulted in the emergence of targeted and immunotherapies. A biopsy that confirms the diagnosis of non-small cell lung cancer is no longer sufficient. Additional tissue will be required for subclassification, immunohistochemistry, PD-L1, next-generation sequencing, and clinical trials [42]. Before considering a procedure, the pulmonologist has to determine not only which procedure is appropriate but also the location to target that would provide the most information required to guide treatment. Close collaboration between cytopathology and medical oncology is therefore of utmost importance [34].

Once a diagnosis of lung cancer is made and the patient is referred to a tumor board for discussion, the role of the pulmonologist does not end there. Central airway obstruction is one of the many severe complications that can result from thoracic malignancies. This complication can lead to severe morbidity and mortality due to sepsis from post-obstructive pneumonia, dyspnea, massive hemoptysis, and other respiratory conditions. Interventional pulmonology plays an important role in central airway obstruction. Central airway obstruction in the context of thoracic malignancy can be due to extrinsic, intrinsic, or mixed compression of the major airways [43]. Bronchoscopic intervention is not always the most appropriate initial treatment. Multidisciplinary discussion with medical and radiation oncology is important because some patients may benefit more from systemic therapy or radiation therapy than from immediate bronchoscopic intervention. Conversely, urgent bronchoscopy may be required to restore airway patency prior to starting systemic therapy [44]. 

Patients with thoracic malignancies frequently have coexisting pulmonary diseases, including chronic obstructive pulmonary disease and interstitial lung disease of smoking-related or non-smoking-related etiologies [45]. Management of these conditions and their complications can be performed by the pulmonologist. Moreover, assessing the patient’s exercise capacity, interpreting pulmonary function tests, managing inhalers, and performing preoperative physiologic risk stratification to evaluate candidacy for lung resection are key functions of the pulmonologist within a multidisciplinary thoracic oncology team [37].

The multitude of cancer pharmaceuticals and radiation therapy has improved outcomes for patients but does not come without its share of complications and side effects. Several immune checkpoint inhibitors can lead to pneumonitis, sarcoid-like granulomatosis, pleural effusion, or exacerbation of obstructive lung disease. Checkpoint inhibitor pneumonitis is a clinically consequential complication and can lead to patient morbidity [46]. A pulmonologist should be involved if there is a concern for this, as the patient may require a bronchoscopy. Stereotactic body radiation therapy, which is a treatment option for lung cancer, can lead to radiation pneumonitis. It can be challenging to differentiate whether the imaging findings are due to progression of malignancy, infection, or pneumonitis from radiation [47]. A multidisciplinary discussion is important with oncology and radiation oncology to review imaging and the patient’s clinical presentation. Bronchoscopy may be required to support one of these diagnoses and can prevent unnecessary discontinuation of effective oncologic therapy.

## 6. Future Directions

Bronchoscopy and pulmonology have become integral to the modern evaluation of pulmonary nodules and the multidisciplinary care of patients with thoracic malignancies. With advancements in technology, bronchoscopes have become thinner, and ultrasound systems have gained improved imaging processing capabilities. Additionally, ancillary imaging technologies, including mobile fluoroscopy and cone-beam CT, have become increasingly portable and integrated into bronchoscopic workflows. Although there have been several key advancements in this decade, more work has to be done for our patients. Despite lung cancer remaining a leading cause of cancer-related death, uptake of LDCT screening remains suboptimal. The majority are diagnosed with lung cancer outside of the screening process, often after developing symptoms or imaging obtained for another reason [48].

The National Lung Screening Trial demonstrated that low-dose computed tomography (LDCT) screening reduced lung cancer mortality by 20% compared with chest radiography [49]. Despite this data, screening uptake remains low and varies according to socioeconomic and health access factors. According to Zgodic et al., cost-related factors and inability to receive care were linked to lower screening rates. They found no statistically significant difference in screening rates when comparing urban and rural groups [50]. Because the underserved population is less likely to receive medical care due to costs, a potential way to improve screening would be to implement mobile lung cancer screening trucks in certain underserved areas. Raghavan et al. described a study using a mobile low-dose CT lung cancer screening unit designed to target the uninsured and rural population. They showed improved screening participation in racial and ethnic minority groups. Among participants eligible for a second annual scan, 66% completed it [51]. Therefore, developing mobile LDCT units can be an effective way to improve screening in vulnerable populations.

A structured nodule program led by a pulmonologist can prevent missed follow-ups, improve adherence to guideline-recommended surveillance, and diagnose lung cancer earlier. Carr et al. looked at incidental lung nodules between 2006 and 2016 and found that a structured radiologist-initiated tracker phrase, along with a lung nodule registry, was associated with more follow-up imaging and a higher likelihood of stage one lung cancer being diagnosed from an incidental nodule [52]. Coronary artery calcium (CAC) scoring CT is increasingly being used for risk stratification, especially since it was incorporated into the American Heart Association guidelines in 2018. Incidental findings such as pulmonary nodules have been seen on coronary calcium CT. The overall incidence of incidental findings is 41%, 18% of which revealed a pulmonary nodule [53]. This creates an opportunity for a pulmonologist-led nodule program to capture early lung cancers that may have otherwise been missed.

Rapid on-site evaluation can help determine whether the current biopsy site has yielded adequate material and whether additional passes or an alternative sampling tool are needed. However, the limited availability of cytopathologists and logistical challenges can make it difficult to use it for every case. Recent advancements in artificial intelligence (AI) are changing the pathology landscape. In a 2024 study, Yan et al. compared the accuracy of interpretation between a cytopathologist and that of machine learning software. The AI system achieved an accuracy of 92.97% for identifying malignant cells during ROSE, compared with 97.15% for the cytopathologist [54]. This comparable accuracy suggests that trained AI models may provide real-time diagnostic feedback during transbronchial sampling. Similarly, digital pathology shifts pathology from a conventional microscope-based examination to digitally scanned slides that can be shared, reviewed, and integrated with AI [55].

During LDCT screening, about 90% of the nodules identified are benign. Therefore, deciding which nodule needs to be biopsied, resected, or monitored becomes the key question facing a pulmonologist. Radiomics is a way to turn medical images into quantifiable data. Instead of looking at a CT and describing a nodule with qualitative adjectives such as spiculated or lobulated, radiomics uses software to extract hundreds of measurable data points from the image, including but not limited to size, volume, density/attenuation, internal heterogeneity, gray level pattern, and margin features [56]. These data points are then entered into a machine learning model to estimate the probability that a nodule is malignant. From the perspective of a lung nodule program, integrating AI can help pulmonologists decide which nodules require a PET scan, biopsy, surgery, or continued surveillance. This could be particularly helpful for indeterminate nodules. Integration of AI to analyze lung nodules should be viewed as a clinical decision support tool rather than replacing physicians’ judgment.

## 7. Conclusions

Lung cancer evaluation has evolved well beyond confirmation of malignancy, and the pulmonologist has become central to this broader diagnostic and therapeutic pathway. Endobronchial and esophageal endosonography have established a minimally invasive foundation for systematic mediastinal and hilar staging when nodal status would alter management, while radial ultrasound, navigational platforms, robotic bronchoscopy, and intraprocedural imaging have expanded the role of bronchoscopy in peripheral pulmonary lesion sampling. Across these domains, procedural success depends not only on reaching the target but also on matching the sampling strategy to lesion anatomy and patient risk, assessing adequacy when possible, and preserving tissue for histologic and molecular characterization. These capabilities deliver their greatest value when integrated into multidisciplinary thoracic oncology care. Continued progress will require not only further procedural innovation but also better systems for screening uptake, incidental nodule follow-up, equitable access to timely diagnosis, and incorporation of emerging tools such as AI and radiomics. As these advances mature, the pulmonologist will remain integral to safe, efficient, and treatment-informative lung cancer care.

## Data Availability

No new data were created or analyzed in this study. Data sharing is not applicable to this article.
